# *Cryptococcus neoformans* Intracellular Proliferation and Capsule Size Determines Early Macrophage Control of Infection

**DOI:** 10.1038/srep21489

**Published:** 2016-02-18

**Authors:** Aleksandra Bojarczuk, Katie A. Miller, Richard Hotham, Amy Lewis, Nikolay V. Ogryzko, Alfred A. Kamuyango, Helen Frost, Rory H. Gibson, Eleanor Stillman, Robin C. May, Stephen A. Renshaw, Simon A. Johnston

**Affiliations:** 1 Department of Infection, Immunity and Cardiovascular Disease, Medical School, University of Sheffield, Sheffield, UK; 2 Bateson Centre, University of Sheffield, Sheffield, UK; 3 Institute of Microbiology and Infection and School of Biosciences, University of Birmingham, Birmingham, UK; 4 School of Mathematics and Statistics, University of Sheffield, Sheffield, UK; 5 NIHR Surgical Reconstruction and Microbiology Research Centre, University Hospitals of Birmingham NHS Foundation Trust, Queen Elizabeth Hospital, Birmingham, UK

## Abstract

*Cryptococcus neoformans* is a significant fungal pathogen of immunocompromised patients. Many questions remain regarding the function of macrophages in normal clearance of cryptococcal infection and the defects present in uncontrolled cryptococcosis. Two current limitations are: 1) The difficulties in interpreting studies using isolated macrophages in the context of the progression of infection, and 2) The use of high resolution imaging in understanding immune cell behavior during animal infection. Here we describe a high-content imaging method in a zebrafish model of cryptococcosis that permits the detailed analysis of macrophage interactions with *C. neoformans* during infection. Using this approach we demonstrate that, while macrophages are critical for control of *C. neoformans,* a failure of macrophage response is not the limiting defect in fatal infections. We find phagocytosis is restrained very early in infection and that increases in cryptococcal number are driven by intracellular proliferation. We show that macrophages preferentially phagocytose cryptococci with smaller polysaccharide capsules and that capsule size is greatly increased over twenty-four hours of infection, a change that is sufficient to severely limit further phagocytosis. Thus, high-content imaging of cryptococcal infection *in vivo* demonstrates how very early interactions between macrophages and cryptococci are critical in the outcome of cryptococcosis.

*Cryptococcus neoformans* is a fungal pathogen of humans that causes life-threatening cryptococcal meningitis in immunocompromised patients, in particular those with advanced AIDS^1^. Cryptococcal meningitis is also found associated with a large variety of other immune deficient states, including in patients on immunosuppressive therapies^2^,^3^, those with hematological malignancy and other more oblique disorders^4^. However, there are a number of unifying features in susceptibility to cryptococcal disease, notably a failure in a pro-inflammatory immune response to primary infection^4^,^5^,^6^.

Primary infection with *C. neoformans* is thought to occur through the lungs where, in the immunocompetent, the first immune cells to encounter cryptococci will most likely be alveolar macrophages. Macrophages alone seem unable to clear infection and an adaptive immune response leading to a self-resolving granuloma is required^7^. This is supported by *in vitro* data demonstrating that *C. neoformans* appears to be able to efficiently parasitise macrophages. *Cryptococcus* survives and proliferates within the phagosome^8^, can escape non-lytically^9^,^10^ and may use macrophages as a ‘Trojan horse’^11^. However, it is not known how host-pathogen interactions at a cellular level determine the outcome of cryptococcosis.

Our understanding of cryptococcosis has been based on a combination of clinical studies, *in vitro* analysis of interactions between cryptococci and isolated mammalian cells, and *in vivo* studies in rodent or leporine hosts. *In vitro* studies offer the advantage of a highly malleable and observable experimental system, but without direct *in vivo* data on disease progression. In contrast, mammalian models provide the opportunity to study progression of infection *in vivo*, but only permit detailed cellular analysis at specific end points.

Most recently this shortfall has been partly addressed using a zebrafish model of cryptococcal disease^12^. The zebrafish is ideally suited for this approach due to a combination of vertebrate immunity, ease of imaging and genetic tractability. Zebrafish have, therefore, been used to model a wide range of human pathogens^13^,^14^,^15^,^16^. Using a similar infection model we have developed a high content imaging methodology that permits a full analysis of cryptococcal and macrophage cell interactions during infection. Using this approach we demonstrate that macrophages are essential for control, but are unable to clear, cryptococcal infection. The ability of macrophages to control infection relies on early phagocytosis, which, in turn, is limited by cryptococcal capsule. Finally, we show that enlargement of cryptococcal capsule is sufficient alone to overcome macrophage phagocytosis *in vivo*, leading to uncontrolled fungal growth and death.

## Results

### A method for the live quantification of macrophage behaviour in response to *Cryptococcus neoformans* infection

**Figure 1:**
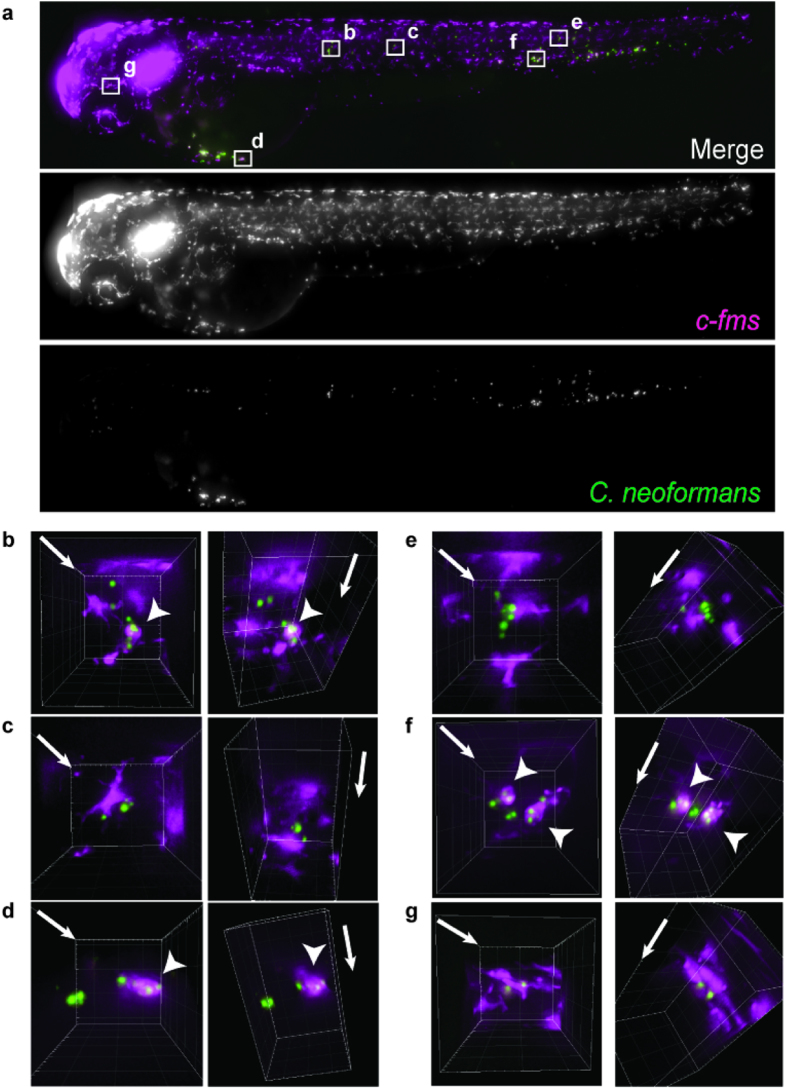
Quantification of macrophage behavior in response to *Cryptococcus* during infection. (**a**) Maximum intensity z-projection of example image data from high content imaging (see Supplementary Fig. S1) of *Tg(fms:Gal4.VP16)i186 ; Tg(UAS:nfsB.mCherry)i149* zebrafish, with mCherry labeled macrophages (magenta), infected with 148 cells of *C. neoformans* strain H99GFP (green), at 2 hours post infection. (**b–d**) Areas boxed in (**a**) enlarged and reconstructed in three-dimensions. Arrowheads indicate intracellular cryptococci. Image pairs represent different views of same volume with arrows indicating z-axis direction. Image grid is 20 μm. Images are representative of a total of 120 infections from n = 3 repeats (40 infections per independent repeat group).

The quantification of macrophage and cryptococcal interactions *in vivo* required sub-cellular resolution imaging throughout the host, while being able to follow the same infections across an extended time period. To achieve this aim we developed repeated high-content live fluorescent imaging, with aligned mounting methods, to image 120 individual blood stream infections of 2 days post fertilisation zebrafish larvae from 3 biological repeats (each biological repeat was performed as an independent group of 40 infected fish; see Supplementary Fig. S1; see Materials and Methods for details of imaging and infection model). Our imaging method was sufficiently resolved in x, y and z to identify both macrophage and cryptococcal cells, their relative location (Fig. 1a; see Supplementary Fig. S2), and whether cryptococci were intracellular (Fig. 1b,d,f; see Supplementary Fig. S2) or extracellular (Fig. 1c,e,g; see Supplementary Fig. S2), throughout the zebrafish. This approach allowed us to track individual fungal cells, providing direct counts for the initial fungal burden that were indistinguishable from parallel cfu counts (see Supplementary Fig. S1). From these data we directly quantified the number of infected macrophages, the number of intracellular cryptococci and total number of cryptococci, at 2 and 24 hours post infection (hpi). Thus, we were able to calculate the number of extracellular cryptococci, the proportion of intracellular cryptococci, the number of cryptococci per macrophage or phagocytic index (PI), and changes in total, intracellular, and extracellular cryptococcal cell numbers between 2 and 24 hours of infection (see Supplementary Data S1). Importantly, since this quantification was carried out in anaesthetized and fully recoverable animals, we were able to place these data in the wider context of subsequent progression of infection on an animal-by-animal basis, to investigate the relationship between macrophage responses and disease.

### Macrophages are essential for the early control of cryptococcal fungemia

**Figure 2:**
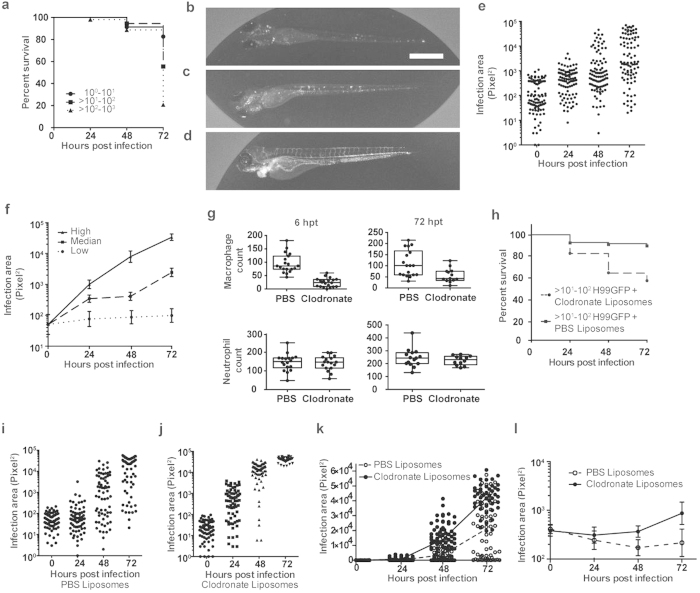
Macrophages are essential for control of cryptococcal fungemia. (**a**) Stratification of survival over 72 hpi from macrophage response data set. Survival is dependent on dose with a large change in survival between 10^1^ and 10^3^. 120 infections from n = 3 (each group contains: 10^0^–10^1^ = 23, >10^1^–10^2^ = 36, >10^2^–10^3^ = 53). (**b–f**) Representative images and quantitation of fungal burden from infections with inocula between >10^1^–10^2^ of *Nacre*-strain zebrafish (100 infections from n = 4). (**b–d**) Fluorescence images of low (**b**), median (**c**) and high (**d**). H99GFP infection of zebrafish at 72 hpi with inocula between >10^1^–10^2^. Scale bar is 500 μm. (**e**) Quantification of fungal burden using area of fluorescent pixels from *C. neoformans* strain H99GFP. Each point is a separate infection with inocula between >10^1^–10^2^, with the same 100 infections over 72 hours. (**f**) Stratification of (**e**) using 2log_10_ boundaries at 72 hpi. Geometric mean with 95% confidence intervals. (**g**) Zebrafish 24 hpf were injected with clodronate or PBS containing liposomes and the numbers of macrophages or neutrophils counted using *Tg(mpeg1:mCherryCAAX)sh378* and *Tg(mpx:GFP)i114* respectively at 6 and 72 hours post treatment. 15 treatments from n = 3. (**h**) Survival of *Nacre*-strain zebrafish infected with inocula between >10^1^–10^2^ of *C. neoformans* strain H99GFP at 48 hpf, following liposome treatment at 24 hpf. P < 0.0001, Log-rank (Mantel-Cox). hazard ratio = 4.5 (logrank; 95% confidence interval 3.2,7.7). 140 and 178 infections from clodronate and PBS groups respectively from n = 3. (**i–k**) Quantification of fungal burden using area of fluorescent pixels from *Nacre*-strain zebrafish infected with inocula between >10^1^–10^2^ of *C. neoformans* strain H99GFP at 48 hpf following treatment with liposomes at 24 hpf. Each point the same 75 infections over 72 hours from n = 3. (**i**) PBS (**j**) Clodronate (**k**) Linear comparison of individual infection and mean fungal burden values with PBS (open circles and dotted line respectively) or clodronate (filled circles and solid line respectively) treatment. Values are the same as presented (**i**) and (**j**). (**l**) Quantification of fungal burden using area of fluorescent pixels from *Tg(mpeg1:mCherryCAAX)sh378* strain zebrafish infected with <5 × 10^1^ of *C. neoformans* strain H99GFP at 48 hpf followed by injection with liposomes at 24 hpi (72 hpf). Points are geometric mean with 95% confidence intervals. The same 25 infections over 72 hours.

As we were able to analyse macrophage responses non-invasively we could also identify time of death in the same experiment. We stratified our infections into three groups according to their initial fungal burden and found a dose response in survival over 72 hpi but saw little difference before this time point (Fig. 2a). In order to provide finer detail on the progression of infection, we used an intermediate dose range (10^1^–10^2^ cryptococci per infection) and measured the changes in fungal burden over 72 hpi by fluorescence microscopy (Fig. 2b–f). We observed that there were a range of infection outcomes, within this narrow dose range, which we could identify as having low, median and high fungal burdens at 72 hpi (Fig. 2b–d, respectively). We quantified this difference by measuring the fungal burden and found that stratification of 72 hpi infection areas at 10^1^–10^2^, 10^2^–10^4^ and >10^4^ square pixels distinguished the same groups (Fig. 2e). Using the 72 hpi divisions we stratified the other time points and found that these groups were present at 24 hpi (Fig. 2f). All three groups showed a significant increase in fungal burden relative to the initial infection but with very large differences in magnitude. The low, median and high groups showed a difference of 2- 10- and 1000-fold increases between 0 and 72 hpi respectively. Having identified differences in the progression of infection in our model we wanted to understand how macrophages contributed to such variability and we used clodronate-containing liposomes to deplete this cell type specifically^17^.

Clodronate treatment 24 hours prior to infection (at 24 hpf) resulted in a rapid depletion in macrophage numbers, which was stable 72 hours after treatment, with neutrophil numbers unaffected (Fig. 2g). Depletion of macrophages using clodronate resulted in a large decrease in survival over 72 hours of infection (Fig. 2h). We measured the fungal burden area and, while PBS-containing liposome treatment showed very similar infection dynamics to our previous analysis (Fig. 2i), macrophage depletion resulted in uncontrolled infection (Fig. 2j). Large differences were already present at 24 hpi and this difference continued to increase at 48 hpi and 72 hpi (difference in mean fungal burden 3.9 × 10^2^, 1.3 × 10^4^ and 2.1 × 10^4^ pixels^2^ respectively; Fig. 2i–k). As depletion of macrophages using clodronate prior to infection resulted in uncontrolled infection we next examined the influence of macrophage depletion later, when control of infection was observed. To establish controlled infection we used a lower initial fungal burden (<5 × 10^1^) where we observed restricted cryptococcal growth over 72 hpi (Fig. 2l) and we depleted macrophages at 24 hpi. We measured the resulting fungal burden and found that following macrophage depletion there was large increase in fungal burden at 48 and 72 hpi (48 hpi 2.3-fold increase, P = 0.0026; 72 hpi, 5.6-fold increase, P = 0.0014; Mann-Whitney test; Fig. 2l). Having established the essential requirement for macrophages in the control of cryptococcal fungemia early in infection, we returned to the question of how the result of the interaction of macrophages with cryptococci influenced the outcome of the infection.

### The majority of cryptococci are intracellular after twenty-four hours post infection

**Figure 3:**
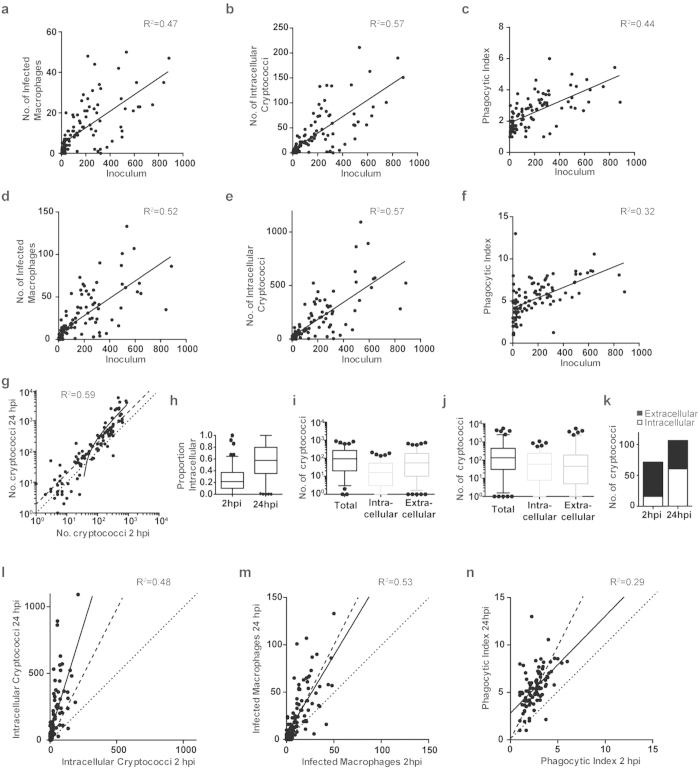
The majority of cryptococci are intracellular at 24 hpi. (**a–f**) Strong positive correlation between inoculum and (**a,d**) number of infected macrophages, (**b,e**) number of intracellular cryptococci and,(**c,f**) phagocytic index at (**a–c**) 2 hpi and (**d–f**) 24 hpi. (**g**). Less than an average two-fold increase in the total number of cryptococci between 2 and 24 hours. Dotted line represents 1 to 1, whereas dashed line represents 2 to 1, relationship. Each point is a separate infection. Solid lines are linear regression, all linear regression P-values < 0.0001. R-squared values are given for each correlation. (**h–k)** Shift to majority of cryptococci being intracellular after 24 hpi. (**h**) The proportion of cryptococci intracellular at 2 and 24 hpi for each infection. Box plot whiskers are 5 and 95 percentiles with outliers plotted. Medians are 0.22 and 0.58 respectively, P < 0.0001 (Mann-Whitney). (**i,j**) Total, intracellular and extracellular cryptococci numbers at 2 hpi (**i**) and 24 hpi (**j**) for each infection. Box plots with whiskers at 5 and 95 percentiles with outliers plotted. (**k**) Median intracellular and extracellular cryptococci numbers at 2 hpi and 24 hpi calculated from data presented in (**I**,**j**). For further descriptive statistics and significance comparisons see Table S1. (**l–n**) Greater increase in number of intracellular cryptococci than the number of infected macrophages between 2 and 24 hpi. Dotted line represents 1 to 1, whereas dashed line represents 2 to 1, relationship. Each point is a separate infection. Solid lines are linear regression, all linear regression P-values < 0.0001. R-squared values are given for each correlation. All data are of 120 infections of *Tg(fms:Gal4.VP16)i186 ; Tg(UAS:nfsB.mCherry)i149* zebrafish with *C. neoformans* strain H99GFP.

We hypothesized that the decreased survival observed at high initial fungal burden in wildtype zebrafish (Fig. 2a) was due to macrophages being overwhelmed, as we had demonstrated for intermediate and low fungal burden via macrophage depletion (Fig. 2j–l). From this hypothesis we predicted that we would see an increase in macrophage responses (i.e. increases in both the number of macrophages containing cryptococci and the number of cryptococci that were intracellular) with increasing inocula up to a threshold where numbers would plateau, corresponding to limited macrophage capacity and control of fungal burden. To test this prediction, we determined the relationship between inoculum and the number of infected macrophages, intracellular cryptococci and the phagocytic index, from our macrophage dataset (Fig 3a–f; see Supplementary Data S1). Analysis of all three measures identified a significant positive linear relationship to inoculum at 2 hpi (linear regression, all P-values < 0.0001; see Fig. 3a–c for individual R^2^-values) but without plateauing at higher inocula. Interestingly, the same relationship was still present at 24 hpi (linear regression, all P-values < 0.0001; see Fig. 3d,e for individual R^2^-values). Thus, while essential for control, the capacity of macrophages to respond to increased numbers of cryptococci, over the range tested, did not appear to be limiting. This was despite the large differences between 2 and 24 hpi in absolute numbers of these values. Examining the total number of cryptococci there was only a small increase between 2 and 24 hours of infection regardless of the initial fungal burden (median increase 0.89; see Supplementary Table S1; Fig. 3g). However, the intracellular and extracellular populations of cryptococci changed dramatically between 2 and 24 hpi, with a large shift to the majority of cryptococci being intracellular at 24 hpi (Fig. 3h). Analysis of the distribution of the intracellular and extracellular numbers from each infection demonstrated that this shift was due to a larger increase in intracellular numbers over static or slightly declining extracellular numbers (Fig. 3h–k; see Supplementary Table S1). In addition, there was a much smaller increase in the numbers of infected macrophages than in the number of intracellular cryptococci, resulting in an increased phagocytic index (Fig. 3l–n). As *C. neoformans* is able to proliferate within macrophages^8^ there are two potential explanations for this observation. Either macrophages that already contain cryptococci are more likely to phagocytose further cryptococci (e.g. due to enhanced levels of activation), or this shift is driven by intracellular proliferation of cryptococci already within macrophages (with subsequent phagocytosis later in infection having only a limited contribution to intracellular burden).

### Intracellular proliferation drives increased numbers of cryptococci at twenty-four hours post infection

**Figure 4:**
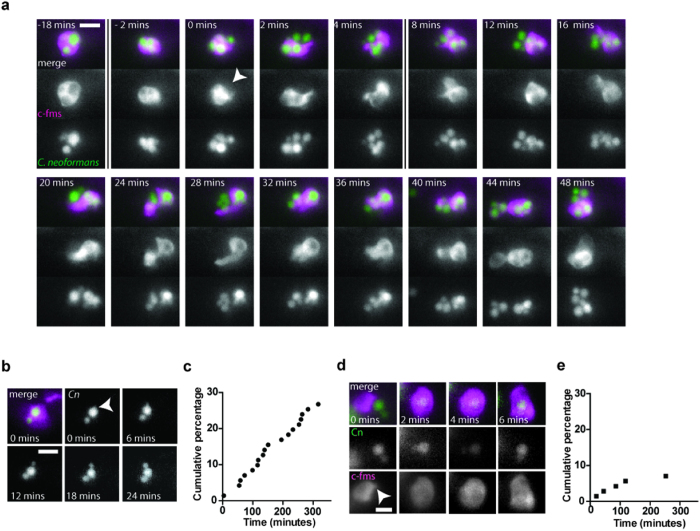
Increase in intracellular numbers of cryptococci is driven by proliferation not phagocytosis. (**a,b,d**) Fluorescent time lapse imaging of parasitism of macrophages. *Tg(fms:Gal4.VP16)i186; Tg(UAS:nfsB.mCherry)i149* zebrafish, with mCherry labeled macrophages (c-fms; magenta), infected with *C. neoformans* strain H99GFP (green). Zebrafish were imaged for 12 hours from 2 hpi. Images were captured every 2 minutes. (**a**) Vomocytosis. Selected frames are presented before and after vomocytic event (0 mins). Arrowhead indicates the formation of concave macrophage membrane with vomocytosis. Vomocytosed cryptococci leave the imaged volume post 48 minutes (see Supplementary Movie S1). Scale bar 10 μm. (**b**) Intracellular proliferation. Selected frames are presented from when bud is first visible (arrowhead). (**c**) Quantification of intracellular proliferation from time lapse imaging. Each occurrence of intracellular budding yeast was counted over 12 hours. Data presented is a cumulative percentage of yeast that budded over the time of observation. (**d**) Phagocytosis. Selected frames are presented from formation of phagocytic cup (arrowhead). (**e)** Quantification of phagocytosis from time lapse imaging. Each occurrence of phagocytosis was counted over 12 hours. Data presented is a cumulative percentage of yeast that were phagocytosed over the time of observation. Quantitation of intracellular proliferation and phagocytosis are from 22 infected macrophages and are representative of n = 5.

To test our predictions we required high temporal resolution as well as spatial resolution such that we could accurately track individual macrophages over time. We used three-dimensional fluorescence time-lapse, taking images every 2 minutes over 12 hours, to identify the exploitation of macrophages by cryptococci. The first important aspect of macrophage parasitism *in vitro* that we examined was vomocytosis, the non-lytic expulsion of cryptococci from macrophages^9^,^10^. The occurrence of vomocytosis has been inferred indirectly *in vivo* but never directly observed^18^. We found that the mechanics of non-lytic expulsion were conserved *in vivo* with a characteristic exocytic, concave membrane during expulsion^19^ (arrowhead Fig. 4a; see Supplementary Movie S1). In addition, we were able to measure the incidence of vomocytosis and found that 5–15% of macrophages expelled cryptococci over 12 hours (Range from n = 9, at least 20 infected macrophages per infection, mean 12%; over 12 hours) and this is consistent with values from mammalian studies^18^,^20^. Similarly, intracellular proliferation could be observed clearly, quantified by the timing of each new visible daughter cell, and occurred regularly over the 12 hours of observation (Fig. 4b,c). In contrast, phagocytosis was much less frequent, with relatively few events in each infection imaged compared to intracellular proliferation (Fig. 4d,e). We therefore concluded that the shift in intracellular numbers of cryptococci was due to intracellular proliferation and not phagocytosis, and we sought to identify the cryptococcal or macrophage phenotype that was limiting phagocytosis after the first hours of infection.

### Cryptococci that are phagocytosed within two hours have small polysaccharide capsules that are absent from the fungal cell population twenty-four hours post infection

**Figure 5:**
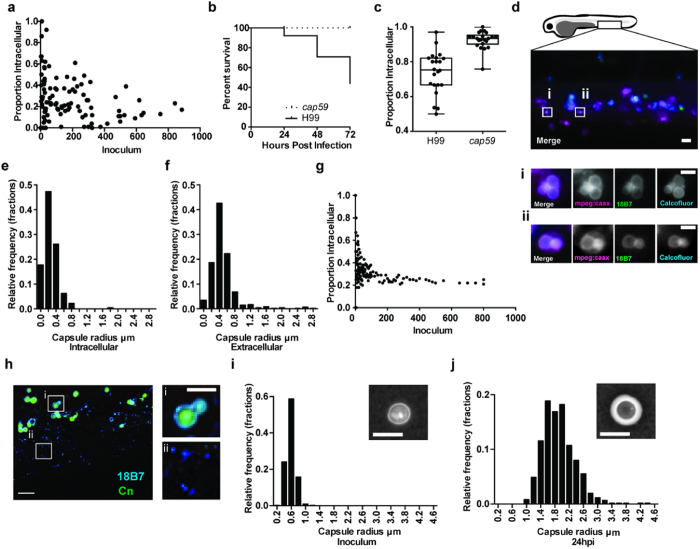
Polysaccharide capsule is smaller on intracellular cryptococci and is greatly enlarged after infection. (**a**) Association between inoculum and proportion of intracellular cryptococci. Each point is a separate infection. P-value 0.0499. 120 infections of *Tg(fms:Gal4.VP16)i186 ; Tg(UAS:nfsB.mCherry)i149* zebrafish with *C. neoformans* strain H99GFP. (**b**) Survival of *AB* strain zebrafish infected with >10^2^–10^3^ *C. neoformans* strain H99 or mutant *cap59*. P < 0.0001, logrank (Mantel-Cox). hazard ratio = 10.1 (logrank; 95% confidence interval 5.6, 18.4). 89 and 81 infections from H99 and *cap59* groups respectively, from n = 8. (**c**) Proportion of intracellular cryptococci 4 hours post infection of *Tg(mpeg1:mCherryCAAX)sh378* with >10^1^–10^2^*C. neoformans* strain H99 or mutant *cap59* labelled with Calcofluor white. Quantitation of 22 infected fish from n = 3 experiments. (**d–f**) *In vivo* measurement of intracellular and extracellular polysaccharide capsule radius. 1025 cryptococci were measured from 50 infections from n = 5 repeats. (**d**) Maximum intensity projection from three dimensional fluorescence imaging of *Tg(mpeg1:mCherryCAAX)sh378* (magenta), that labels macrophage membranes, infected with inocula between >10^1^–10^2^ of *C. neoformans* strain H99 labeled for polysaccharide capsule (green) and cell wall (cyan). Boxed areas are enlarged in i and ii, and are single z-sections. Scale bar is 20 μm in left image and 5 μm in i and ii. (**e,f**) Intracellular cryptococci have smaller polysaccharide capsules. Relative frequency histograms of capsule radius for intracellular (**e**) and extracellular (**f**) cryptococci. (**g**) Output of probability model using relative numbers within macrophages from (**e**,**f**) to calculate proportion of intracellular cryptococci at different inocula given random input (**h**) Cryptococcal capsule is enlarged and shed at 24 hpi. Fixed tissue of *AB* strain zebrafish infected with >10^2^–10^3^ *C. neoformans* strain H99GFP (green) at 24 hpi labeled with antibody to capsular polysaccharide (cyan). Scale bar 10 μm. Boxed areas are enlarged in i and ii. Scale bar 5 μm. (**i,j)** Cryptococcal capsule size greatly enlarged 24 hpi. Relative frequency histograms of capsule radius for inoculum (**i**) and 24 hpi (**j**) cryptococci isolated from *AB* strain zebrafish infected with >10^2^–10^3^ of *C. neoformans* strain H99. Inset panels are example India ink stained samples. Scale bar 5 μm. 615 cryptococci were measured in 12 infections from n = 3.

Unlike the numbers of infected macrophages and intracellular cryptococci, the proportion of cryptococci that were intracellular at 2 hpi was only very weakly related to the inoculum (linear regression P = 0.0499, R^2^ = 0.035; Fig. 5a) i.e. over the range of inocula observed, the proportion of cryptococci phagocytosed was stable (median proportion phagocytosed 0.25; 95% CI (0.21, 0.29)). This suggested that there was a consistent subset of the cryptococcal population that was not phagocytosed.

In order to understand how modulating phagocytosis influenced the outcome of infection we attempted to reduce the phagocytosis of cryptococci by blocking likely uptake pathways. Soluble mannan and glucan have been shown to block the uptake of fungal pathogens by macrophages^21^. However, we found that co-injection of these molecules was insufficient to alter uptake of cryptococci by macrophages in our model (see Supplementary Fig. S3). The polysaccharide capsule of *C. neoformans* is its defining clinical microbiological feature and has been reported to have a broad range of immunosuppressive activities including preventing phagocytosis^22^,^23^,^24^. To define the role of capsule in the pathogenesis of cryptococcosis in our model we used the *cap59* mutant that has severely compromised capsule formation^25^. Infection with the *cap59* mutant resulted in zero mortality over 72 hours of infection despite using a dose that caused greater than 50% mortality in the parental H99 strain (Fig. 5b). In addition, analysis of the uptake of the *cap59* mutant by macrophages demonstrated that almost all cryptococci were intracellular (Median proportion intracellular 0.93; Fig. 5c). Therefore, we hypothesized that differences in cryptococcal capsule size were sufficient to define the limitation of phagocytosis, both in the primary phase following infection (Fig. 5a) and later in a secondary phase (>2 hpi) where phagocytosis was largely curtailed (Fig. 4f).

We predicted that 2 hpi macrophages would contain cryptococci with smaller capsules than extracellular cryptococci. To measure the size of the cryptococcal capsule *in vivo* accurately, we combined immunofluorescence labeling of the capsule and staining of the cell wall immediately prior to infection. Live measurement of capsule sizes 2 hpi demonstrated that intracellular cryptococci had capsules only about half the radius of extracellular cryptococci capsules (extracellular mean = 0.49 μm, median = 0.43 μm; intracellular mean = 0.26 μm, median = 0.24 μm; P < 0.0001, Mann-Whitney; Fig. 5d–f; see Supplementary Fig. S4). Furthermore, the relative frequency of the different intracellular and extracellular capsule sizes was sufficient to closely model the distribution of phagocytosis values we had quantified from our previous infections (Fig. 5g).

We next investigated the capsule at 24 hpi, the time point after which we had proposed that capsule was limiting phagocytosis. As changes in capsule could not be observed live in the same way as early in infection we immuno-labeled fixed tissue and found that by 24 hpi cryptococcal capsule was enlarged, with shed capsular material clearly present in surrounding tissue (Fig. 5h). We quantified capsular size *ex vivo* and found that there was a large, and highly consistent, difference in capsule size (Fig. 5i,j; see Supplementary Fig. S4). This large and rapid shift in capsule size potentially explained the limitation of phagocytosis later in infection, given the relative ability of macrophages to phagocytose cryptococci with different capsule sizes and as there was almost no overlap between the capsule size of the initial fungal burden and the fungal cell population at 24 hpi.

### Enlargement of capsules of initial fungal burden limits phagocytosis and restricts macrophage control of cryptococci

**Figure 6:**
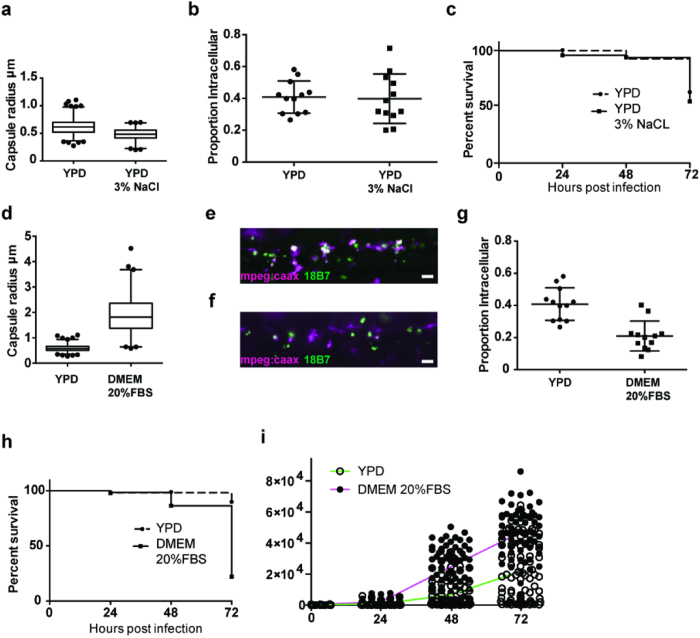
*In vitro* recapitulation of large polysaccharide capsule prevents macrophage phagocytosis *in vivo*. (**a**) Reduction of cryptococcal capsule *in vitro.* P < 0.001, Mann-Whitney; Medians YPD = 0.62 μm, YPD 3%NaCl = 0.48 μm. 632 (YPD) and 345 (YPD 3% NaCl) infections, India ink staining from n = 3. (**b**) Proportion of intracellular cryptococci 2 hpi of *Tg(mpeg1:mCherryCAAX)sh378* with >10^1^–10^2^ *C. neoformans* strain H99GFP grown in YPD or YPD 3% NaCl. Each point represents a separate infection from n = 4 plotted with median and standard deviation. (**c**) Survival of *AB*-strain zebrafish infected with >10^2^–10^3^ of *C. neoformans* strain H99GFP grown in YPD or YPD 3% NaCl (P = 0.38, logrank (Mantel-Cox)). 55 (YPD) and 66 (YPD 3% NaCl) infections, from n = 3. (**d**) Induction of cryptococcal capsule *in vitro.* P < 0.001, Mann-Whitney, Medians YPD = 0.58 μm, DMEM 20% FBS = 1.81 μm. 513 (YPD) and 255 (DMEM 20% FBS) India ink staining from n = 3. (**e,f**) Example maximum intensity projection from three-dimensional fluorescence imaging of *Tg(fms:Gal4.VP16)i186;Tg(UAS:nfsB.mCherry)i149* (magenta) infected with >10^1^–10^2^ of *C. neoformans* strain H99GFP (green) 2 hpi. Intracellular yeast appear white due co-localisation of both colours (**e**) YPD inoculum. (**f**) DMEM 20% FBS inoculum. (**g**) Proportion of intracellular cryptococci 2 hpi of *Tg(mpeg1:mCherryCAAX)sh378* with >10^1^–10^2^ *C. neoformans* strain H99GFP grown in YPD or DMEM 20% FBS. Each point represents a separate infection from n = 4 with median and standard deviation. (**h**) Survival of *AB*-strain zebrafish infected with >10^1^–10^2^ of *C. neoformans* strain H99GFP grown in YPD or DMEM 20% FBS. P < 0.0001, logrank (Mantel-Cox). hazard ratio = 8.2 (logrank; 95% confidence interval 6.5, 20.0). 57 and 59 infections from YPD and DMEM 20% FBS groups respectively, from n = 3. (**i**) Linear comparison of quantification of fungal burden using area of fluorescent pixels from *Nacre*-strain zebrafish infected with between >10^1^–10^2^ of *C. neoformans* strain H99GFP grown in YPD or DMEM 20% FBS. Individual infection and mean fungal burden values with H99GFP grown in YPD (open circles and green line respectively) or H99GFP grown in DMEM 20% FBS (filled circles and magenta line respectively) treatment. Each point is a separate infection; the same 100 (YPD) or 97 (DMEM 20% FBS) infections followed over 72 hours from n = 3. Box plots are whiskers at 5 and 95 percentiles with outliers plotted.

We tested how the modulation of capsule size influenced the outcome of infection by using *in vitro* culture methods that modified capsule size prior to infection. *In vitro* culture with NaCl was sufficient to significantly reduce capsule size^26^ (Fig. 6a) but the reduction in capsule was not sufficient to increase uptake by macrophages or survival of infection in contrast to the capsule mutant *cap59* (Fig. 6b,c). The increase in capsule size observed *in vivo* can be induced *in vitro* using mammalian serum, increased ambient CO_2_ concentration, limited iron availability increased pH and temperature^27^,^28^,^29^. We tested combinations of temperature, mammalian growth media (MGM), mammalian serum and nutrient starvation for induction of cryptococcal capsule similar to those that we had observed *in vivo* in zebrafish. A combination of MGM, serum and 37 °C gave a capsule size much larger than growth in rich media and a distribution very similar to that seen at 24 hpi (median = 1.87 μm vs. 1.81 μm MGM and 24 hpi respectively, P = 0.24, Mann-Whitney; Fig. 6a).

Using induced capsule cultures we were able to probe the effect on macrophage phagocytosis of enlarged capsules at the initiation of infection. When zebrafish were infected with capsule-enlarged *C. neoformans* there were significantly fewer intracellular cryptococci (Fig. 6b,c). Quantification of intracellular cryptococci by live fluorescence imaging showed that there was an approximately two-fold reduction in the proportion of intracellular cryptococci (Fig. 6d). This reduction in intracellular cryptococci had a dramatic impact on survival with almost 80% mortality and an 8.2 hazard ratio in comparison to non-induced cultures (logrank, 95% confidence interval 6.5, 20.0; Fig. 6e) and on fungal burden (difference at 72 hpi 2.8 × 10^4^, P = <0.0001, Mann-Whitney). We therefore concluded that increased capsule size at 24 hpi was sufficient to prevent macrophage control of cryptococcal infection and led to uncontrolled fungal growth and death.

## Discussion

Here we have presented analysis of macrophage and cryptococcal cell interactions that enables analysis at a cellular level *in vivo*, non-invasively and over the course of infection. High quality imaging is the mainstay of the zebrafish model and our imaging methodology allows whole organismal imaging of interactions of the host immune system and pathogen cells, non-invasively throughout a vertebrate host. Single plane illumination microscopy (SPIM) and related technologies^30^,^31^ offer similar benefits, and have the potential for even higher spatial resolution, but our approach has the advantage of being able to image tens to hundreds of infections, in parallel, over a relatively short time period. This means that large datasets can be generated and subjected to robust statistical analysis. A major limiting step to be overcome is that image analysis of such datasets remains largely a manual task. This is due to the complex nature of the detection and segmentation of immune and pathogen cells, and the amount of computation required, as even a single infected zebrafish, at a single time point amounts to over a billion voxels.

A potential limitation to the zebrafish model for studying human infection is that the zebrafish are maintained at 28 °C as opposed to 37 °C. Nevertheless, zebrafish have been have been used successfully to study a wide range of human bacterial and fungal pathogens^13^,^14^,^15^,^16^,^32^. Host temperature has a clear role in pathogenicity of fungal pathogens and is one reason why *Cryptococcus neoformans* is a significant pathogen of humans when other cryptococcal species are not^33^. However, given that *C. neoformans* appears able to infect such a wide range of animal species, with very different thermoregulation, it is likely that pathogenesis is not dependent of host body temperature^34^. The trehalose pathway of *C. neoformans* has been shown to be a requirement for growth at 37 °C and the trehalose pathway mutant Δ*tps1* is avirulent in mice^35^. However, the Δ*tps1* mutant was also avirulent in zebrafish at 28 °C and the nematode *Caenorhabditis elegans* at 25 °C^12^,^35^. Similarly, here we have demonstrated that changes in cryptococcal capsule during infection of zebrafish agree with mammalian studies and are therefore not dependent on body temperature alone.

We sought to understand the behavior of phagocytes during unresolved infection, when the immune system must clear growing yeast cells, by injection of cryptococcoci into the circulation. This does not model the likely route of human infection of a very low inoculum into the alveolar space but our route of infection directly relates to our aim of studying the early events following dissemination of *C. neoformans*. We note that other models of infection are possible with the zebrafish, whereby pathogens are, for example, introduced to single restricted tissue sites or, most intriguingly, to the swim bladder, an enclosed air/liquid interface of epithelial/mesothelium tissue^36^,^37^,^38^.

We have demonstrated that, while macrophage phagocytosis of cryptococci contributes to control of infection, in the absence of adaptive immunity, the developing immune system of zebrafish larvae alone (that can be considered a model of vertebrate innate immunity) cannot clear cryptococcal infection. Our model permitted unparalleled detail in the progression of cryptococcal infection and we able to observe distinct outcomes of infection, within narrow ranges of initial fungal burden, that appeared stochastic. The progression of cryptococcal infection was very sensitive to differences in dose and, even within small ranges, this may be sufficient to explain largely the differences in infection progression. However, there were numerous examples of initial fungal burden being independent of severity (see Supplementary Data S1). Therefore a stochastic model (where effect is independent of initial fungal burden but the probability of an effect is not) best fits our current understanding of the progression of cryptococcal infection and is likely also to be applicable to other aspects e.g. the dissemination to the central nervous system.

Nevertheless, macrophages are essential for control, as their depletion had a catastrophic effect on any restriction of fungal burden, even when neutrophil numbers were unaffected. This was also recently demonstrated in zebrafish using a transient knockdown of the Spi-1 transcription factor^12^. In addition, depletion of macrophages once infections were controlled still led to increased fungal burden, suggesting they continue to play a critical role even after the initial onset of disease. Previous studies that have depleted macrophages in a mouse lung model of cryptococcosis have shown differing results. Intratracheal or intranasal administration of clodronate liposomes resulted in decreased or unaltered lung fungal burden in three different mouse strains^39^. However, this approach may not deplete macrophages and dendritic cells in surrounding tissues that may be able to compensate for local loss^40^. In contrast, the depletion of CD11c-expressing macrophages and dendritic cells using a diphtheria toxin-sensitive transgenic caused no observable difference in lung fungal burden 4 days post infection (dpi) but resulted in considerable mortality at 5 dpi^40^. Our results provide an explanation for this finding, by demonstrating that there can be very rapid changes in fungal burden following the loss of macrophage control. Interestingly, macrophage depletion late in infection has been demonstrated to be protective in dissemination of *C. neoformans* and provides evidence for the role of the parasitism of macrophages in dissemination during cryptococcosis^11^.

Our approach also provides validation for multiple aspects of macrophage parasitism by *C. neoformans* that have previously been characterized *in vitro*. Vomocytosis was originally identified in mammalian macrophages and has been demonstrated in environmental amoeboid hosts^9^,^41^. However, this study represents the first time vomocytosis has been directly observed and quantified *in vivo*. The impact of vomocytosis during progression of cryptococcosis is unknown; does vomocytosis protect host macrophages from parasitism or is it fundamental to the dissemination of *C. neoformans* in cryptococcal meningitis? A previous study was able to infer the occurrence of vomocytosis *in vivo* but required the isolation of macrophages from the lung following infection^18^. Since this is not a limitation for our zebrafish model, there is now the potential to be able to identify when and how vomocytosis contributes to the pathogenesis of cryptococcal infection.

Similarly, intracellular proliferation is a significant *in vitro* phenomenon but how it contributes to the progression of infection is not known. In the related pathogen *C. gattii*, intracellular proliferation closely correlates with virulence in mice and humans but this has not been observed for *C. neoformans*^42^,^43^,^44^. We have shown that not only can intracellular proliferation be directly observed *in vivo*^12^ but that intracellular proliferation is the principal factor driving the shift in the proportion of cryptococci that were intracellular at twenty-four hours post infection. Thus, the previously described, apparently, higher proportion of phagocytosis observed at 13 hpi is likely due to intracellular proliferation not phagocytosis^12^. Interestingly, a similar result was observed in *C. neoformans* infection of mouse lungs between 2 and 8 hours but using a high fungal inoculum, perhaps explaining the much earlier peak in intracellular cryptococci compared to our study^45^. High intracellular growth will be protective to the host early in infection as there will be limited tissue damage from extracellular growth. Early limitation of damage to host tissue by intracellular growth may also contribute to pathogenesis as this will reduce pro-inflammatory immune signaling permitting cryptococcal infection to become established.

However, whilst intracellular proliferation proceeds rapidly *in vivo*, there is little change in extracellular yeast numbers early in infection, reflecting the need for extracellular yeast to adapt to the host environment prior to the rapid growth seen later in infection. Gene expression analyses during lung infection have demonstrated a similar ‘rest’ period for extracellular yeast during adaptation^44^,^46^. Intriguingly, this does not appear to be the case for intracellular yeast in macrophages, as they are able to proliferate almost immediately. The more rapid changes in intracellular yeast gene expression profile may perhaps be part of a host adaptation to dormancy^44^.

The infectious propagule of human cryptococcosis is most likely a basdiospore or yeast cell desiccated to the extent that it is small enough to reach the deep structures of the lung^47^. In either case the capsule will be very thin or absent, unlikely to inhibit phagocytosis, and thus capsule thickness will likely not be a factor for initial infection in the lungs. Using live imaging of double capsule and cell wall labeled cryptococci *in vivo* we have shown that, even for small capsules following growth in rich media, cryptococcal capsule thickness is a determinant of phagocytosis *in vivo*. The ability of capsular polysaccharide size to interfere with phagocytosis is one of the earliest reported findings in cryptococcal pathogenesis^22^ but the mechanism by which capsule inhibits phagocytosis, especially opsonic uptake, remains to be proven^48^. Where *C. neoformans* is not controlled and cleared, both intracellular and extracellular cryptococci will rapidly develop enlarged capsules^45^,^49^. Our data shows that polysaccharide capsule enlargement occurs rapidly after infection, with larger capsules seen as early as two hours post infection. Cryptococcal capsule enlargement has been described in a zebrafish at 5 dpi^12^, however, we find that similarly enlarged capsules are present by 1 dpi. The enlargement of capsule we see during the first 24 hpi infection and infection with *C. neoformans* with capsules similarly enlarged *in vitro*, is sufficient to restrict phagocytosis severely. Thus, the restriction of phagocytosis for any given dose will increase the number of extracellular cryptococci and increase the likelihood of uncontrolled infection. Similar experiments have not been proven to be feasible in the mouse lung infection model due to the inability of large encapsulated yeast to enter deep into the lung^26^. Infection with an acapsular mutant of *Cryptococcus* results in similar fungal burden to a wildtype cryptococcal strain^12^ and we found that an acapsular mutant was avirulent and almost completely limited to residing intracellularly. *In vitro* restriction of capsule did not significantly alter the outcome of any of these measures, presumably as the reduction in capsule size was not sufficient to increase phagocytosis and once in the host capsular polysaccharide was induced as with the normally cultured strain. We therefore presume that damage to host tissues is associated with extracellular growth as the acapsular is avirulent despite considerable intracellular growth and the enhanced mortality in our infections with cryptococci with *in vitro* induced polysaccharide capsules.

Cryptococci have evolved both to evade phagocytosis and survive within phagocytes^50^. Here we have demonstrated a mechanism by which this combination of behaviors, which have presumably evolved to avoid predation in the environment^50^, are particularly destructive in the progression of human cryptococcal infection. The number of fungal organisms in the initial inoculum of human infection is likely to be very low. Therefore, in healthy individuals, with competent adaptive immune responses, it is very likely that all cryptococci will be intracellular and will be killed following the pro-inflammatory activation of macrophages. However, in the absence of such responses the combination of survival within macrophages and the inhibition of phagocytosis by cryptococcal capsule will lead to the uncontrolled progression of infection.

## Materials and Methods

### Ethics statement

Animal work was carried out according to guidelines and legislation set out in UK law in the Animals (Scientific Procedures) Act 1986, under Project License PPL 40/3574. Ethical approval was granted by the University of Sheffield Local Ethical Review Panel.

### Fish husbandry

We used the *Nacre*^51^ and *AB* strains as our wild type strains, as indicated in the figure legends. Two macrophage (*Tg(fms:Gal4.VP16)i186; Tg(UAS:nfsB.mCherry)i149*^52^ and *Tg(mpeg1:mCherryCAAX)sh378)* and one neutrophil (*Tg(mpx:GFP)i114*^53^) fluorescent transgenic zebrafish lines were used. Zebrafish strains were maintained according to standard protocols^54^. Adult fish were maintained on a 14:10-hour light/dark cycle at 28 °C in UK Home Office approved facilities in the Bateson Centre aquaria at the University of Sheffield.

### Transgenic line generation

We generated a transgenic zebrafish with fluorescently labeled macrophage membranes using the CAAX motif to cause the prenylation of mCherry. The mpeg1:mCherryCAAX expression vector was generated using the Tol2 Kit Gateway system^55^ by recombining pME-mCherryCAAX with pDestTol2pAG2, p3E-PolyA and the *mpeg1* promoter entry clone^56^. The resulting expression vector was used to generate *Tg(mpeg1:mCherryCAAX)sh378* as described previously^54^.

### *C. neoformans* culture

The *C. neoformans* variety *grubii* strain H99, its GFP-expressing derivative H99GFP and the polysaccharide capsule production mutant *cap59* were used in this study^57^. 2 ml YPD (reagents are from Sigma-Aldrich, Poole, UK unless otherwise stated) cultures were inoculated from YPD agar plates and grown for 18 hours at 28 **°**C, rotating horizontally at 20 rpm. Cells were pelleted at 3300 g, washed twice with PBS (Oxoid, Basingstoke, UK) and resuspended in 2 ml PBS. In addition, as the *cap59* mutant tended to form cell clumps, the washed *cap59* cells were incubated at room temperature for thirty minutes and only the top 50 μl, that contains only single or doublet cells, used for infections (other strains used in parallel to the *cap59* mutant were treated identically). Washed cells were counted with a hemocytometer and used as described below.

### *C. neoformans* capsule induction or restriction

Cultures of H99 GFP were washed and resuspended in PBS as described above. For capsule induction 0.5 ml of washed cells for each culture was centrifuged and suspended in 2 ml of Dulbecco’s Modified Eagle’s Medium (DMEM; D5546) with 20% heat inactivated foetal bovine serum (FBS; F9665) to act as the inducing agent. For capsule restriction 0.5 ml of washed cells for each culture was centrifuged and suspended in 2 ml of YPD with 3% w/v NaCl to act as the restricting agent. Control cultures were also prepared using YPD, and DMEM alone. Cultures were grown for a further 24 hours either rotating at 28 °C or in an orbital shaker at 250 rpm at 37 °C. Cultures were then washed three times in PBS to remove residue of the different growth media. Induction or restriction of capsule of all cultures was assessed using India ink staining described below.

### Zebrafish model of *C. neoformans* infection

The volume of counted, washed cryptococci was calculated to give the required inoculum in 1 nl, and this volume was pelleted at 3300 g. Pellets were resuspended in autoclaved 10% Polyvinylpyrrolidinone (PVP), 0.5% Phenol Red in PBS (PVP is a polymer that increases the viscosity of the injection fluid and prevents settling of microbes in the injection needle^58^). For co-injection of mannan and laminarin these were added at 100 μg/ml. Embryos were anesthetised at 2 days post fertilization (dpf) by immersion in 0.168 mg/mL tricaine in E3, transferred onto a microscope slide and covered with 3% methyl cellulose in E3 for injection. Two 0.5 nl boluses were injected into the yolk sac circulation valley. Zebrafish were transferred to fresh E3 to recover from anaesthetic. Any zebrafish that had visible damage from the injection or where the injections were not visually confirmed by the presence of Phenol Red were removed. Zebrafish were maintained at 28 °C.

### High content imaging method

Infected zebrafish were anesthetized by immersion in 0.168 mg/mL tricaine E3 and mounted in agar channels for imaging. Channels were made by adding 200 μl of 1% agar (Cat. No. 05039) in E3 containing 0.168 mg/mL tricaine into glass-bottomed, 96-well plates (Porvair sciences, Wrexham, UK). Channels were cut in cooled agar using GelX4 tips (Geneflow, Staffordshire, UK). Mounted embryos were imaged on a Nikon Ti-E with a CFI Plan Apochromat λ 10X, N.A.0.45 objective lens, a custom built 500 μm Piezo Z-stage (Mad City Labs, Madison, WI, USA) and using Intensilight fluorescent illumination with ET/sputtered series fluorescent filters 49002 and 49008 (Chroma, Bellow Falls, VT, USA). Images were captured with Neo sCMOS, 2560 × 2160 Format, 16.6 mm x 14.0 mm Sensor Size, 6.5 μm pixel size camera (Andor, Belfast, UK) and NIS-Elements (Nikon, Richmond, UK) using the following settings: **1.** GFP, filter 49002, 10 ms exposure, gain 4 **2.** mCherry, filter 49008, 10 ms exposure, gain 4. Each zebrafish was imaged as three contiguous fields of view that were assigned from bright-field images. 80 z sections, 5 μm apart, were captured in each channel and each position in that order. Each biological repeat contained 40 infected zebrafish, with 3 multi-channel z stacks per fish. The microscope was enclosed in a humidified, 28 °C, environmental chamber (Okolabs, Pozzuoli, Italy). After imaging larvae were recovered in fresh E3 and returned to a new numbered 96-well plate.

### Processing of high content imaging

High content images were not processed for analysis except adjustment of look-up-tables to temporarily increase local contrast. For presentation in Fig. 1a images were projected in the z-plane using the maximum intensity pixel method. Three-dimensional reconstructions in Fig. 1 and Fig. S2 were performed using Imaris (Bitplane, Zurich, Switzerland).

### Macrophage response data set

Eight of the 120 infections were censored at 2 hpi and removed from the analysis, seven due to having an initial fungal burden of zero and one due to the larvae being damaged by the transfer to the imaging plate. A further 2 infections were censored at 24 hpi due to an inability to make the counts due to the quality of the imaging files. Any censored or missing values are indicated by ‘NA’ in the data tables and were not included in any relevant analysis. The following calculations were performed to obtain values for derived counts: number of extracellular cryptococci = total number of Cryptococci − number of intracellular cryptococci; proportion of intracellular cryptococci = number of intracellular Cryptococci/total number of cryptococci; phagocytic index = number of intracellular Cryptococci/number of infected macrophages; change in cryptococcal numbers between 2 and 24 hpi = (number of cryptococci at 24 hpi-number of cryptococci at 2 hpi)/number of cryptococci at 2 hpi.

### Imaging and colony forming units (CFU) counts

Imaging and CFU counts were compared from the same infections. Zebrafish 2 hpi infection were imaged as described above, followed by manual dissociated of individual larvae with microcentrifuge pestles in 200 μl dH_2_0 (this will lyse host cells while leaving fungal cells intact^42^). Dissociates were plated on YPD agar and incubated at 28 °C for 48 hours before counting.

### Measurement of fungal burden area

Zebrafish were imaged in 96-well plates using Nikon Ti-E with a CFI Plan Achromat UW 2X N.A. 0.06 objective lens, using Intensilight fluorescent illumination with ET/sputtered series fluorescent filters 49002 (Chroma, Bellow Falls, VT, USA). Images were captured with Neo sCMOS, (Andor, Belfast, UK) and NIS-Elements (Nikon, Richmond, UK). Images were exported as tif files and further analysis performed in ImageJ (Schneider *et al.*, 2012). Images were individually cropped to remove the side of the 96-well or any bright debris or noise within the well. Pixels above the intensity corresponding to *C. neoformans* strain H99GFP were selected using a threshold. The same threshold was used for all images. Thresholded images were converted to binary images and the number of pixels counted using the ‘analyse pixel’ function.

### Macrophage depletion using clodronate liposomes

Clodronate or PBS liposomes (Clodronateliposomes, Amsterdam, The Netherlands) were diluted 1:1 in 10% Polyvinylpyrrolidinone (PVP), 0.5% Phenol Red in PBS. 1 nl was injected into embryos 1 dpf or 3 dpf (after removal from the chorion) as described above. Specific depletion was confirmed using macrophage *(Tg(mpeg1:mCherryCAAX)sh378)* and neutrophil (*Tg(mpx:GFP)i114*) transgenic zebrafish.

### Time lapse imaging

Time lapse imaging was performed as described for high content imaging with the following adjustments: Zebrafish larvae were mounted in 0.8% low melting point agarose (Cat No. A9414) in E3 containing 0.168 mg/mL tricaine. Images were captured with CFI Plan Apochromat λ 20X, N.A.0.75 objective lens, 10 z-sections 2.5 μm apart, with Perfect Focus system, every 2 minutes for 12 hours.

### Survival

Survival was assessed by presence or absence of heart-beat. Statistical analysis was performed as described in the text and figure legends.

### Live measurement of *C. neoformans* capsule size

*C. neoformans* at 1 × 10^7^/ml were labeled with monoclonal antibody 18B7 (a gift from Arturo Casadevall) as described previously^19^,^59^. 18B7 labeled cryptococci were then labeled with 2.5 μg/ml FITC secondary antibody and 15 μg/ml fungal cell wall stain Calcofluor white (Cat No. 18909) for 45 mins at 28 °C, rotating horizontally at 20 rpm (the *cap59* mutant and H99GFP were similarly labeled with Calcofluor white for imaging of cryptococcal uptake in Fig. 5c). Labeled cryptococci were re-counted and injections were performed as above. Imaging was performed as for time lapse except at a single time point 2 hpi and using, in addition, a 31000v2 fluorescent filter for Calcoflour white staining (Chroma, Bellow Falls, VT, USA). NIS Elements (Nikon, Richmond, UK) was used to measure capsule radius by subtracting cell wall diameter from capsule diameter and halving.

### Antibody staining

Zebrafish larvae were fixed at room temperature in 4% formaldehyde for 30 minutes rocking, washed three times in PBS with 0.1% Triton-X and incubated rocking for 10 mins. Washing was repeated twice more. Fixed larvae were incubated in 5 μg/ml 18B7 primary antibody in 500 μl 0.1% Triton-X solution rocking at 4 °C for 16 hours. Following primary antibody staining, larvae were washed as above. They were then incubated with 5 μg/ml CF350 secondary antibody (Cat No. SAB4600222) in 500 μl 0.1% Triton-X rocking at room temperature for 2 hours, washed as above and mounted on microscope slides with 7 μl Mowiol solution^19^ under 13 mm coverslips.

### Probability model for prediction of proportion of intracellular cryptococci

We wrote a Microsoft Excel (2011 v14.5.3) spreadsheet containing a probability model that calculated whether an individual cryptococci was phagocytosed or not based on its capsule size. The relative proportions of capsule sizes presented in Fig. 5c,d were used to calculate the probability of phagocytosis given such a capsule size. Thus, using a random number input, probability of phagocytosis (β) was calculated from β = IF(A < 0.12,0.71,IF(A < 0.45,0.55,IF(A < 0.786,0.22,IF(A < 0.94,0.11,0.13)))) where A is a random number between 0 and 1. Successful phagocytosis (ϕ; i.e. cryptococcal cell was intracellular) was then scored by: ϕ = IF(A < β,1,0). ϕ was calculated for each cryptococcal cell in an infection and summed to give the proportion of cryptococci intracellular. The model was run repeatedly over the range of initial fungal burdens observed observed and plotted (Fig. 5e) as measured data (Fig. 5a).

### India ink assay for cryptococcal capsule size

For staining of *C. neoformans* cultures, equal volumes (2 μl) of cell suspension and India ink (Winsor and Newton, London, UK) were mixed on a microscope slide and mounted under a 13 mm coverslip. For staining of cryptococci following infection, zebrafish larvae were dissociated with microcentrifuge tube pestles in 20 μl PBS, pelleted at 16300 g for 5 mins, resuspended in 3 μl PBS, 3 μl of India ink added and mounted as above. India ink samples were imaged on Leica HC upright microscope with phase contrast PL APO 100 × 1.4NA objective lens and images captured with ProgRes C14 camera and software. ImageJ was used to measure capsule radius by subtracting cell body diameter from total diameter.

### Statistical analysis

Statistical analysis was performed as described in the results and figure legends. We used Graph Pad Prism 6 (v6.0b) for statistical tests and plots.

## Additional Information

**How to cite this article**: Bojarczuk, A. *et al.* *Cryptococcus neoformans* Intracellular Proliferation and Capsule Size Determines Early Macrophage Control of Infection. *Sci. Rep.* **6**, 21489; doi: 10.1038/srep21489 (2016).

## Supplementary Material

Supplementary Information

Supplementary Data S1

Supplementary Movie S1
