# Supplementary material for: Cryptococcus neoformans Intracellular Proliferation and Capsule Size Determines Early Macrophage Control of Infection
Source: Sci Rep. 2016 Feb 18;6:21489. doi: 10.1038/srep21489 (PMC4757829; doi:10.1038/srep21489)
Supplement: Supplementary Information [file srep21489-s1.pdf]

Supplementary Information

Cryptococcus neoformans Intracellular Proliferation and Capsule Size Determines Early Macrophage Control of Infection

Aleksandra Bojarczuk[a,b], Katie A. Miller[a,b], Richard Hotham[a,b], Amy Lewis[a,b], Nikolay V. Ogryzko[a,b], Alfred A. Kamuyango[a,b], Helen Frost[c#], Rory H. Gibson[a,b], Eleanor Stillman[d], Robin C. May[c,e], Stephen A. Renshaw[a,b] and Simon A. Johnston[a,b*]

Supplementary Figure S1

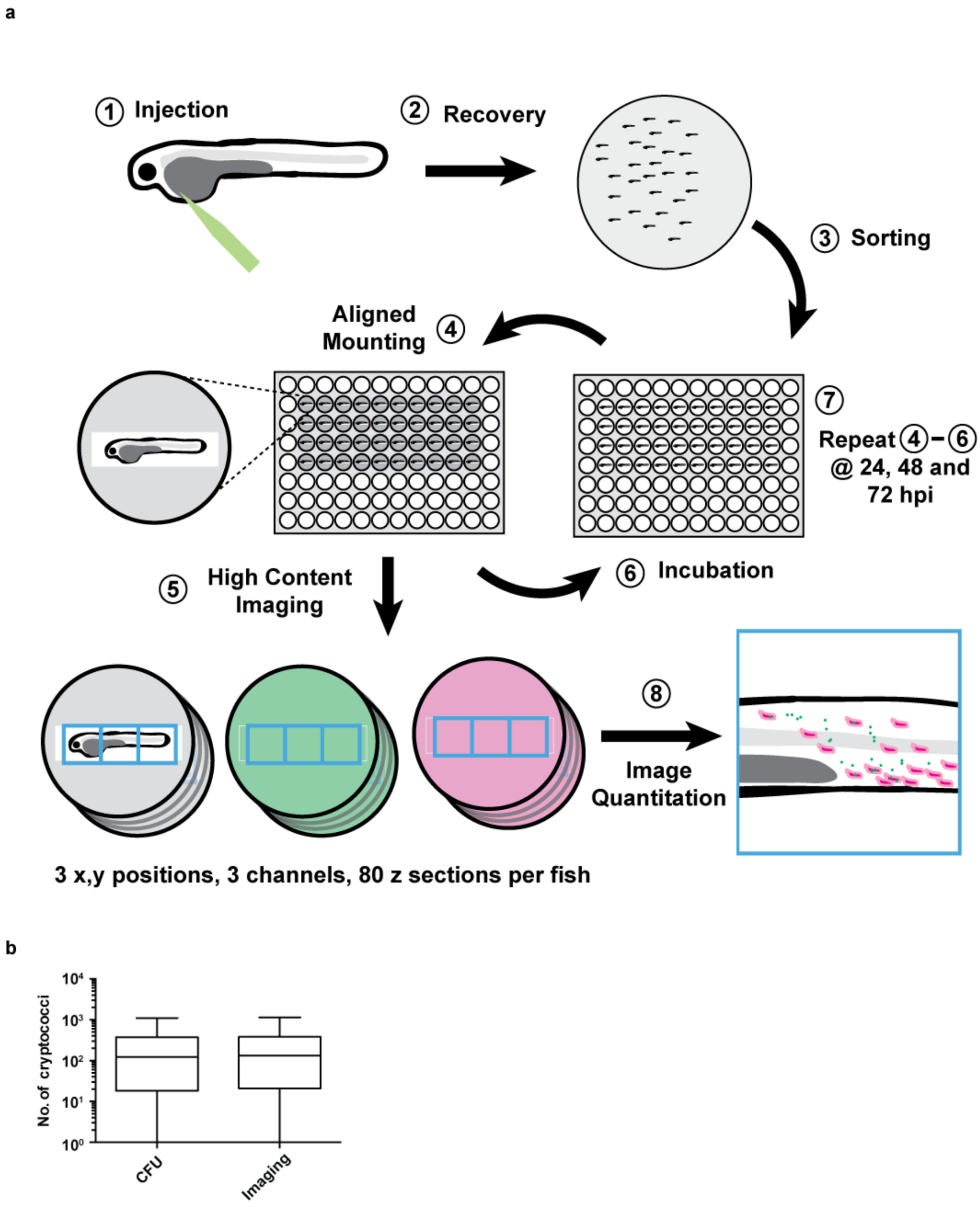

Method pipeline for high content imaging of zebrafish *Cryptococcus* infection model. (a) 1. Anesthetized zebrafish were injected a 2 dpf with H99GFP C. neoformans. **2.** Zebrafish were recovered from anesthetic and any injured individuals removed. **3.** Forty zebrafish were randomly selected and sorted into 96-well plates per repeat. **4.** For imaging, zebrafish were

anesthetized and aligned in agar channels in a second 96-well plate. **5.** Three dimensional image data sets were captured and the zebrafish were recovered from the anesthetic and returned, to the same plate for incubation. **6.** Infected zebrafish were incubated at 28°C **7.** Steps 4-6 were repeated at 24, 48 and 72 hpi. **8.** Each data set was manually counted to generate the values in Table S1. **(b)** Comparison of the values obtained from image counts and CFU plating. The number of H99GFP were counted from images described above, followed by tissue dissociation and plating to establish CFU counts. Pooled counts of 120 infections from n=3 repeats.

Supplementary Figure S2

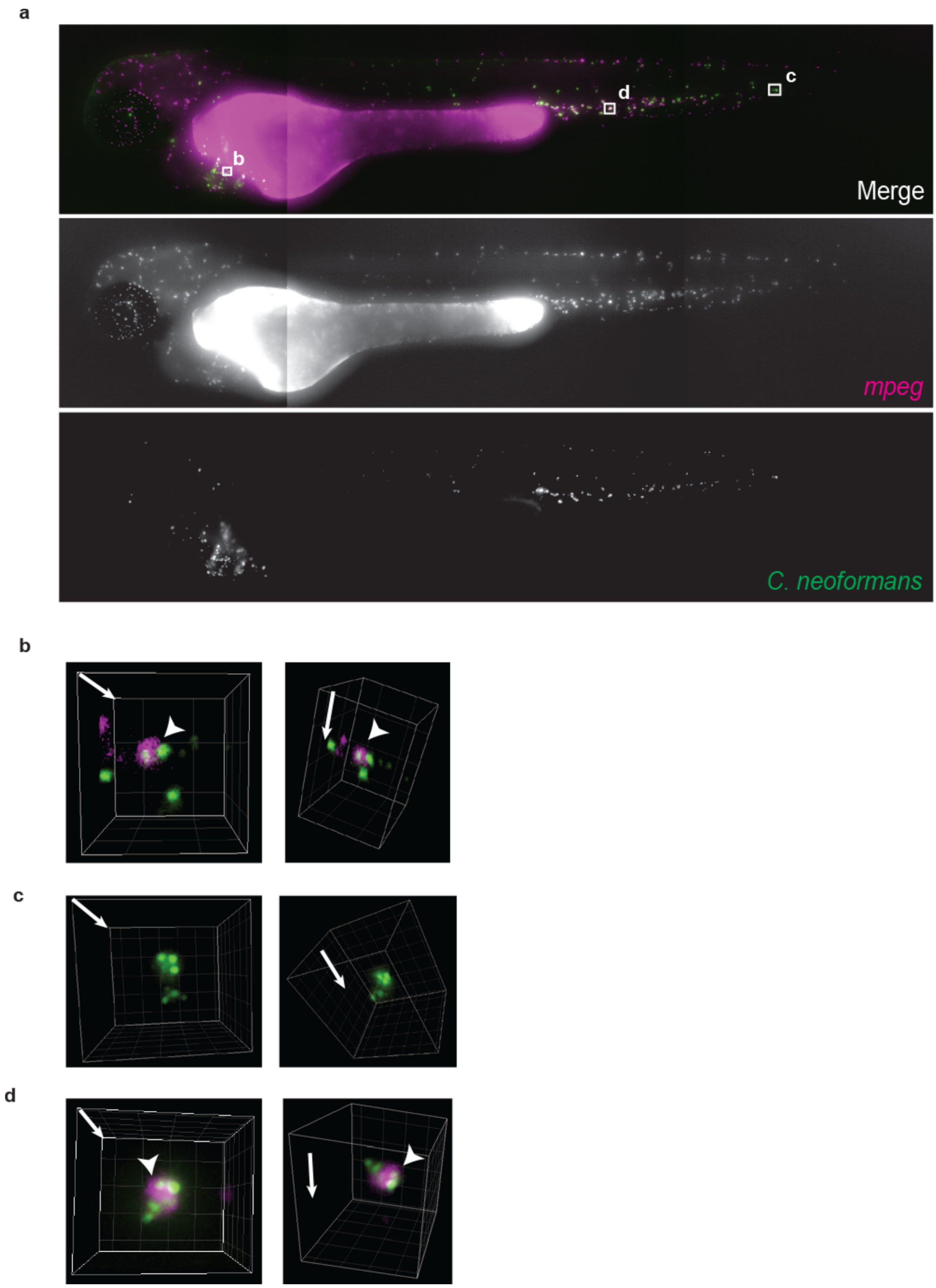

Quantification of macrophage behavior in response to Cryptococcus during infection using *mpeg* macrophage marker. (a) Maximum intensity z-projection of example image data from high content imaging of *Tg(mpeg1:mCherryCAAX)sh378* zebrafish, with mCherry labeled macrophages (magenta), infected with 208 cells of *C. neoformans* strain H99GFP (green), at 2 hours post infection. **(b,c,d).** Areas boxed in (a)

enlarged and reconstructed in three-dimensions. Arrowheads indicate intracellular cryptococci. Image pairs represent different views of same volume with arrows indicating z-axis direction. Image grid is 20µm.

Supplementary Figure S3

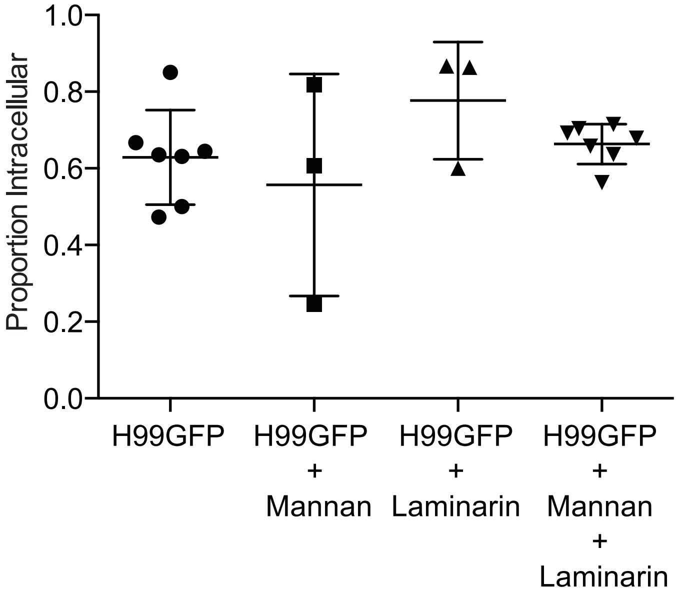

Co-injection of 100 µg/ml mannan and/or laminarin did not inhibit uptake of cryptococci by macrophages in zebrafish. Proportion of intracellular cryptococci 2 hours post infection of *Tg(mpeg1:mCherryCAAX)sh378* with >10^1-10^2 *C. neoformans* strain H99GFP alone or co-injected with 100 µg/ml mannan and/or laminarin. Each point represents a separate infection plotted with median and standard deviation.

Supplementary Figure S4

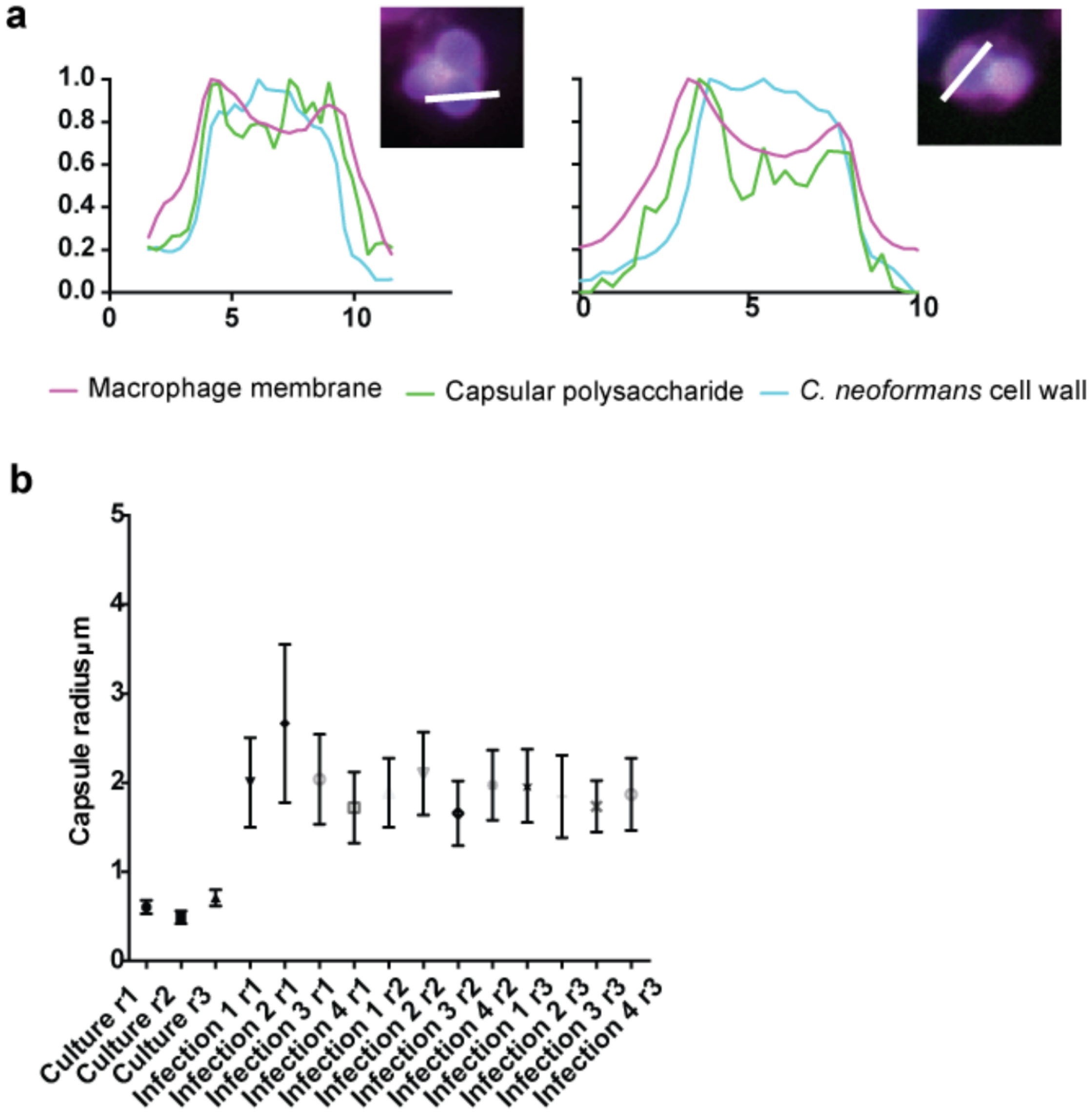

Polysaccharide capsule is smaller on intracellular cryptococci and is greatly enlarged after infection. A. Macrophage membrane, capsule and cell wall relative positions can be accurately measured from intensity profiles. Normalised pixel intensity (to the brightest pixel on each path) for the three channels was measured along the lines drawn and plotted. **B.** Mean and standard deviation of capsule radius measurements of each control and infection presented in Fig. 5i,j.

Supplementary Table S1.

	Median	Mean	P-value 2 hpi vs. 24 hpi
Number of Cryptococci at 2 hpi	93	168	0.037
Number of Cryptococci at 24 hpi	135	505	
Number of Intracellular Cryptococci at 2 hpi	16	34	<0.0001
Number of Intracellular Cryptococci at 24 hpi	61	154	
Number of Extracellular Cryptococci at 2 hpi	56	134	0.427
Number of Extracellular Cryptococci at 24 hpi	46	354	
Increase in Total Numbers of Cryptococci	0.89	1.42	N/A
Increase in Intracellular Numbers of Cryptococci	3.05	5.34	N/A
Increase in Extracellular Numbers of Cryptococci	-0.012	0.61	N/A

Descriptive statistics and significance tests for the number of cryptococci at 2 and 24 hpi. Values derived from Data S1. Mann-Whitney test used for significance comparison.

Additional Legends

Supplementary Data S1 (see separate file).

Macrophage response data set. Comma separated values. 'NA' represents missing data values either because of direct censoring (see Materials and Methods) or because missing or zero counted values prevented the calculation of derived values. For the calculation of other values see Materials and Methods.

.

Supplementary Movie S1.

Movie of cell presented in Figure 4a. Images are at 5 frames per second.
